# 13-Plex DeAla Isobaric Reagents for High-Throughput Proteome Quantification

**DOI:** 10.1021/acs.analchem.5c03910

**Published:** 2025-10-06

**Authors:** Peng-Kai Liu, Ting-Jia Gu, Shuling Xu, Alexander Nassar, Zicong Wang, Hung-Yu Chiang, Danqing Wang, Lingjun Li

**Affiliations:** † Biophysics Graduate Program, 5228 University of Wisconsin-Madison, Madison, Wisconsin 53705, United States; ‡ School of Pharmacy, University of Wisconsin–Madison, Madison, Wisconsin 53705, United States; § Department of Chemistry, University of Wisconsin–Madison, Madison, Wisconsin 53706, United States; ∥ Lachman Institute for Pharmaceutical Development, School of Pharmacy, University of Wisconsin–Madison, Madison, Wisconsin 53705, United States; ⊥ Wisconsin Center for NanoBioSystems, School of Pharmacy, University of Wisconsin–Madison, Madison, Wisconsin 53705, United States

## Abstract

Isobaric labeling techniques are widely used in mass spectrometry-based quantitative proteomics to enable the simultaneous analysis of multiple samples. However, commercial isobaric tags are expensive due to complex synthesis and costly reagents, limiting their use in large-scale studies. Here, we introduce a novel, cost-effective diethylalanine-based isobaric reagent (DeAla), synthesized using diethylated alanine and β-alanine with *N*-hydroxysuccinimide. The DeAla tag offers several advantages, including improved peptide fragmentation, enhanced protein identification, and competitive pricing. We optimized labeling efficiency and collision energy parameters, demonstrating that DeAla-labeled peptides produce more backbone fragmentation ions and higher XCorr values compared to peptides labeled with *N*,*N*-dimethyl leucine (DiLeu) tags. By selectively incorporating stable isotopes, we expanded the multiplexing capacity to 13-plex without increasing structural complexity, achieving baseline resolution in Orbitrap MS/MS acquisition at 60k resolution. Comparative proteomic analyses of two cancer cell lines demonstrated that DeAla labeling outperformed DiLeu tags and showed comparable performance to label-free approaches in terms of protein and peptide identification. Additionally, DeAla provided accurate and reproducible quantification across a dynamic range with minimal technical variability. Overall, the 13-plex DeAla reagents are cost-effective, high-performance isobaric tagging tools that enhance peptide fragmentation and protein identification while ensuring high quantification accuracy, making them valuable for complex quantitative proteomic analyses.

## Graphical abstract

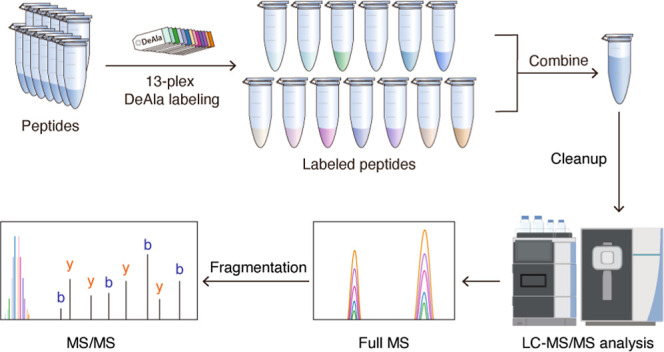

## Introduction

Mass spectrometry-based proteomics has emerged as an essential tool for unraveling the complex molecular mechanisms underlying cellular biology.^1–4^ The ability to quantify proteins with high precision and sensitivity has opened new avenues for studying cellular dynamics, biomarker discovery, and the intricate interactions within protein networks.^5–8^ Quantitative proteomics can be divided into label-free and label-based approaches. Label-free quantitation (LFQ) does not require additional chemical labeling and quantifies protein abundance by directly comparing precursor ion intensities across samples.^9–11^ However, LFQ suffers from variability across runs and lacks the multiplexing capability needed for high-throughput studies.^12,13^ Alternatively, chemical isotope labeling techniques, such as stable isotope labeling by amino acids in cell culture (SILAC) and dimethyl labeling, introduce stable isotopes into samples to create distinguishable mass shifts at the MS1 level.^14–18^ While these methods enhance quantitation accuracy by reducing variability between runs, they increase MS1 spectral complexity and are typically limited in multiplexing capacity.^19,20^ To address these limitations, isobaric labeling has emerged as a powerful approach that enables the simultaneous quantification of multiple samples by tagging peptides with chemically identical but isotopically distinct labels.^21,22^ The MS1 signal of an isobarically labeled peptide appears as a single peak, and upon fragmentation, distinct reporter ions are released and detected at the MS2 level. This allows for relative quantification of each sample based on the intensity of the reporter ions. This approach significantly enhances throughput, reduces variability, and improves data reliability for quantitative proteomics.^23–26^

Commercial isobaric tags, such as tandem mass tags (TMT) and isobaric tags for relative and absolute quantitation (iTRAQ), are widely employed in quantitative proteomics, providing powerful tools for precise and high-throughput analysis of complex biological samples.^27–30^ However, a notable drawback of commercial isobaric tags is their high cost, driven by complex synthesis and expensive reagents. This cost barrier limits accessibility for many research laboratories, especially when conducting large-scale quantitative studies. Therefore, there is a significant demand for the development of cost-effective isobaric tags. Researchers have developed alternative, affordable tags, including deuterium isobaric amine-reactive tags (DiART) and isobaric tags (IBT) for the relative quantitative analysis of proteomes.^31–33^ Our lab has developed cost-effective isobaric reagents, dimethyl leucine (DiLeu) and dimethyl alanine (DiAla), as economical alternatives to commercial isobaric tags.^34,35^ Multiplex DiLeu reagents have been widely used in high-throughput proteomic studies for applications such as disease biomarker discovery, investigations of cellular responses, and analyses of protein interaction networks.^36–39^

Despite their cost benefits, the labeling protocols for the triazine ester tags, including DiLeu and DiAla, require additional steps, such as carboxylic acid activation prior to labeling and the use of strong cation exchange columns to remove residual reagents. These extra steps can lead to sample loss, impeding optimal protein and peptide coverage. Another critical factor in isobaric tagging is the efficiency of peptide fragmentation. Tags that enhance peptide fragmentation and improve the signal quality of fragment ions significantly contribute to the accuracy of protein identification and quantification.^40,41^ Recent studies have shown that alanine-based isobaric tag labeling generates more abundant fragmentation ions, whereas DiLeu labeling produces more intense reporter ions but suppresses peptide fragment ions, resulting in fewer identifications with DiLeu labeling.^35^

In this study, we introduce a new alanine-based labeling reagent, DeAla, synthesized using diethylated alanine and beta-alanine with *N*-hydroxysuccinimide (NHS), enabling direct peptide labeling without the need for additional activation. DeAla offers several advantages, including enhanced fragmentation and improved sensitivity for fragment ions, increased protein identification in quantitative proteomics, and significant cost savings. The DeAla tag was synthesized in three steps with high yield. Labeling efficiency and collision energy parameters were optimized using DeAla-labeled tryptic peptides of MDA-MB-231 cells. We then evaluated their impact on peptide fragmentation and found that DeAla labeling produced more backbone fragmentation ions and higher XCorr values compared to DiLeu in shared labeled tryptic peptides from bovine serum albumin (BSA) and MDA-MB-231 cells. We extended the comparison to proteomics experiments in two cancer cell lines: MDA-MB-231 (breast cancer) and PANC-1 (pancreatic cancer), to evaluate DeAla, DiLeu, TMT, and label-free approaches. To expand the multiplexing capacity of isobaric reagents, 13-plex DeAla reagents were achieved without increasing structural complexity by exploiting mass defects generated through the selective incorporation of ^13^C, ^15^N, and ^2^H stable isotopes in the reporter group.^42^ The resulting 13 reporter isotopologues differed by mass intervals of 5.84 mDa or 6.32 mDa, allowing for baseline resolution in Orbitrap MS/MS acquisition at 60k resolution (at *m*/*z* = 200), ensuring precise and accurate quantification. To demonstrate the performance of these reagents, we used the Thermo Scientific Exploris 480 Orbitrap mass spectrometer to accurately quantify mixtures of 13-plex DeAla-labeled MDA-MB-231 breast cancer cell tryptic peptides. We compared the labeling performance of 12-plex DiLeu and 13-plex DeAla in terms of peptide and protein identification numbers, as well as the impact of the tags on fragmentation. Overall, the cost-effective 13-plex DeAla labeling reagents demonstrated superior multiplexing capacity and fragmentation efficiency, offering enhanced peptide and protein identification compared to 12-plex DiLeu, while maintaining high quantification accuracy and reproducibility in complex proteomic analyses.

## Experimental Section

### Material

Heavy isotopic l-alanine and β-alanine were purchased from Cambridge Isotope Laboratories (Tewksbury, MA). Optima LC/MS grade acetonitrile (ACN), formic acid (FA), and water were obtained from Fisher Scientific (Pittsburgh, PA). ACS grade acetaldehyde, acetaldehyde-^13^C_2_, acetonitrile, acetone, dichloromethane (DCM), methanol (MeOH), *N*,*N*-dimethylformamide (DMF), 1.0 M triethylammonium bicarbonate buffer (TEAB), 4-methylmorpholine (NMM), 4-(4,6-dimethoxy-1,3,5-triazin-2-yl)-4-methylmorpholinium tetrafluoroborate (DMTMM), *N*,*N*′-diisopropylcarbodiimide (DIC), *N*-hydroxysuccinimide (NHS), sodium cyanoborodeuteride (NaBD_3_CN), sodium cyanoborohydride (NaBH_3_CN) were purchased from Sigma-Aldrich (St. Louis, MO). TMTzero was purchased from Thermo Fisher (Waltham, MA). *N*,*N*,*N*′,*N*′-Tetramethyl-*O*-(*N*-succinimidyl)­uronium tetrafluoroborate (TSTU) was purchased from TCI (Portland, OR). Mass spectrometry-grade trypsin was purchased from Promega (Madison, WI). All reagents were used without further purification.

### Cell Culture

Human epithelial adherent breast carcinoma cell line MDA-MB-231 (ATCC # HTB-26) and human epithelial adherent pancreatic carcinoma cell line PANC-1 (ATCC #CRL-1469) were purchased from American Type Culture collection (ATCC) and used for in vitro proteomics study. The cells were maintained as a monolayer in Dulbecco’s modified Eagle’s medium (DMEM) (Gibco #11995065), supplemented with 10% fetal bovine serum (FBS) (Gibco #10437028), 100 U/ml of penicillin, 100 μg/mL of streptomycin (Gibco #5140122) in a 37 °C humidified 5% CO_2_ incubator. For cell passage, 80–90% confluent cells were washed with phosphate buffered saline (PBS) and trypsinized with Trypsin–EDTA (0.05%) (Gibco #25300062) for 5 min. The cells were split at 1:4 subcultivation ratio for regular maintenance and the complete medium was renewed every 2 to 3 times/week. The cells were counted by using the Bright-Line Hemacytometer (Hausser Scientific #1492). To demonstrate DeAla labeling in proteomic analyses, MDA-MB-231 and PANC-1 cells were cultured to 80% confluence, harvested by trypsinization, and quenched with complete DMEM. The cells were collected by centrifugation at 450*g* for 5 min and washed three times with PBS. The cell pellets were lysed in a buffer containing 8 M urea, 50 mM HEPES, a protease inhibitor cocktail (Roche #04693159001), and a phosphatase inhibitor cocktail (Roche #04906837001). Lysis was enhanced by two rounds of sonication (30% amplitude, 5 s on/10 s off for 1 min) using a probe-type sonicator (Fisher Scientific #FB120 with a probe #CL-18). Lysates were centrifuged at 21,000*g* for 10 min, soluble protein concentration was determined by the BCA protein assay kit (Thermo Scientific #23225). The lysates were then prepared for subsequent reduction and alkylation steps.

### Protein Extract Digestion

Proteins were dissolved in 8 M urea with 50 mM HEPES, reduced with 10 mM TCEP at 37 °C for 30 min, and alkylated with 20 mM iodoacetamide (IAA) in the dark for 45 min. The urea concentration was then diluted with 50 mM HEPES buffer to less than 1 M. Trypsin was added at a 1:50 (w/w) enzyme-to-protein ratio, and the mixture was incubated at 37 °C for 16 h. The digestion reaction was quenched with 10% trifluoroacetic acid (TFA) to a final concentration of 0.3% TFA. Peptides were desalted using Sep-Pak C18 cartridges (Waters, Milford, MA) and dried in vacuo.

### 13-Plex DeAla Labeling

DeAla labeling was performed by adding the labeling solution at a 10:1 tag-to-peptide ratio (by weight) and vortexing at room temperature for 2 h. For example, 1 mg of DeAla tag in 30 μL ACN was added to 100 μg of peptide dissolved in 70 μL of 50 mM TEAB. To quench the reaction, 5% hydroxylamine was added to achieve a final hydroxylamine concentration of 0.25%, followed by vortexing for 10 min. Peptides labeled with different channels were combined in two specific ratios: 1:1:1:1:1:1:1:1:1:1:1:1:1 and 1:2:4:8:12:16:8:16:12:8:4:2:1. The combined sample was dried in vacuo, cleaned using SCX SpinTips (TT200SEA tip, PolyLC containing 12 mg PolySULFOETHYL A beads, 20 μm, 300 Å), and either desalted using Sep-Pak C18 cartridges (Waters, Milford, MA) or purified by high-pH (HpH) fractionation according to the manufacturer’s protocol.

### 12-Plex DiLeu Labeling

DiLeu labeling was performed by adding the labeling solution at a 10:1 tag-to-peptide ratio (by weight) and vortexing at room temperature for 2 h, as reported previously. For example, 1 mg of DiLeu tag was reconstituted in 100 μL of DMF and activated with DMTMM and NMM at 0.6× molar ratios for 45 min. The activated DiLeu tag was added to 100 μg of peptides dissolved in 20 μL of 0.5 M TEAB, and the reaction mixture was vortexed for 2 h. To quench the reaction, 5% hydroxylamine was added to achieve a final hydroxylamine concentration of 0.25%, followed by vortexing for 10 min. Peptides labeled with different channels were combined in a 1:1:1:1:1:1:1:1:1:1:1:1 ratio. The combined sample was dried in vacuo, cleaned using SCX SpinTips, and either desalted using Sep-Pak C18 cartridges (Waters, Milford, MA) or purified by high-pH (HpH) fractionation according to the manufacturer’s protocol.

### HpH Fractionation

HpH fractionation was performed on a Waters Alliance e2695 HPLC system using a C18 column (Phenomenex, 150 mm × 2.1 mm, 5 μm, 100 Å) operating at a flow rate of 0.2 mL/min. Mobile phase A consisted of 10 mM ammonium formate in water at pH 10, and mobile phase B consisted of 10 mM ammonium formate in 90% ACN at pH 10, with the pH of both phases adjusted using ammonium hydroxide. Separation was carried out using the following gradient: 1% B (0–5 min), 1–40% B (5–50 min), 40–60% B (50–54 min), 60–70% B (54–58 min), 70–100% B (58–59 min), 100% B (59–74 min). Fractions were collected every 2 min, staring from 6 to 60 min, and nonadjacent fractions were concatenated into four tubes for proteomic analysis.

### LC–MS/MS Analysis

Labeled peptide samples were analyzed on a Thermo Scientific Q Exactive mass spectrometer coupled to a Waters nanoAcquity UPLC system or Thermo Scientific Orbitrap Exploris 480 mass spectrometer coupled to a Vanquish Neo UHPLC system. Samples were dissolved in water with 0.1% formic acid and then loaded onto a 75 μm inner diameter self-fabricated microcapillary column packed with 15 cm of C18 beads (1.7 μm, 130 Å, Waters). On the Q Exactive mass spectrometer, Mobile phase A was composed of water with 0.1% formic acid. Mobile phase B was composed of ACN with 0.1% formic acid. Separation was performed using a gradient elution of 4–30% mobile phase B over 120 min at a flow rate of 300 nL/min. For precursor MS scans, 350–1400 *m*/*z* were collected at a resolving power of 35k (at *m*/*z* = 200) with automatic gain control (AGC) target of 7 × 10^4^ and a maximum injection time of 50 ms. Tandem MS scans were performed using HCD with 28% normalized collision energy at a resolving power of 35k and the top 10 precursors were selected for HCD analysis. Tandem MS scans were acquired with 99 *m*/*z* first mass, an AGC target of 1 × 10^5^, a resolution of 70k (at *m*/*z* = 200), an intensity threshold of 1 × 10^4^, an isolation window of 1.2 *m*/*z* units, a maximum injection time of 250 ms. Precursors were subjected to dynamic exclusion for 45 s. On the Exploris 480 mass spectrometer, mobile phase A was composed of water and 0.1% formic acid. Mobile phase B was composed of 80% ACN and 0.1% formic acid. Separation was performed using a gradient elution of 5–32% mobile phase B over 120 min at a flow rate of 300 nL/min. For precursor MS scans, 350–1400 *m*/*z* were collected at a resolving power of 120k (at *m*/*z* = 200) with AGC target of 5 × 10^5^ and a maximum injection time of 50 ms, followed by MS/MS of the most intense precursors for 3 s. Tandem MS spectra were performed using HCD with 32% normalized collision energy at a resolving power of 60k (at *m*/*z* = 200). Tandem MS scans were acquired with 99 *m*/*z* first mass, an AGC target of 1 × 10^5^, an intensity threshold of 1 × 10^4^, an isolation window of 1 *m*/*z* units, a maximum injection time of 120 ms. Precursors were subjected to dynamic exclusion for 30 s. Each sample was acquired in technical duplicates.

### Data Analysis

Protein identification and quantification from mass spectrometry (MS) data were conducted using Proteome Discoverer (version 2.5, Thermo Scientific). The raw data files were searched against the UniProt *Homo sapiens* reviewed database (July 22, 2024) using the Sequest HT algorithm. Trypsin was specified as the enzyme, and two missed cleavages allowed. Fixed modifications included DeAla labeling on peptide N-termini and lysine residues (+203.14728 Da) and carbamidomethylation of cysteine residues (+57.02146 Da). Oxidation of methionine residues (+15.99492 Da) was set as a variable modification. Peptide spectral matches (PSMs) were validated on the basis of *q*-values to 1% false discovery rate (FDR) using Percolator. Reporter ion quantification in MS2 spectra was performed using Proteome Discoverer with an integration tolerance of 20 ppm for the most confident centroid. Only the PSMs containing all 13 reporter ions were considered. Reporter ion intensities were exported to Excel, and isotopic interference correction was performed according to the previously reported method.^34^

## Results and Discussions

### Design and Synthesis of the DeAla Reagent

The DeAla isobaric tag shares a common structure with other isobaric reagents, comprising a reporter group, a balance group, and an amine-reactive group. We designed a novel alanine-based tag using NHS as the amine-reactive group, diethylated alanine as the reporter group, and β-alanine as the balance group. The synthesis of the DeAla tag involves reductive diethylation, β-alanine conjugation, and carboxylic acid esterification with NHS (Figure S1). First, alanine undergoes diethylation with acetaldehyde and NaBH_3_CN to form diethylalanine. This intermediate then undergoes a two-step coupling with TSTU and β-alanine, followed by activation with DIC and NHS to yield the DeAla tag in its NHS ester form, with an overall isolated yield of 70% over three steps. This synthetic route enables cost-effective and large-scale tag production. DeAla tags can directly label primary amine groups on the lysine side chain and the N-terminus of peptides. Upon higher-energy collision dissociation (HCD) or collision-induced dissociation (CID), the attached tags fragment into diethyl immonium reporter ions with *m*/*z* values separated by at least 5.8 mDa.

### Optimization of Labeling Efficiency and Collision Energy for the DeAla Reagent

The labeling efficiency of the DeAla reagent was evaluated by labeling tryptic peptides derived from MDA-MB-231 cells. The percentage of labeled N-terminal and lysine residues was determined by calculating the proportion of labeled peptides relative to the total number of identified peptides, including both labeled and unlabeled species. Labeling efficiency was assessed across a range of DeAla tag-to-peptide weight ratios from 1:1 to 40:1. As shown in Figure S2, labeling efficiency was 63.3% at a 1:1 ratio and increased with higher tag-to-peptide ratios, achieving complete labeling at ratios above 10:1. Therefore, a 10:1 tag-to-peptide ratio was selected as the optimal condition for peptide labeling. Next, we optimized the HCD normalized collision energy (NCE) using a Q Exactive and an Exploris 480 mass spectrometers under different NCE values (Figure S3). The results indicated that an NCE of 28 on a Q Exactive and an NCE of 30 on an Exploris 480 mass spectrometer yielded the highest numbers of protein and peptide identifications for DeAla-labeled peptides.

### Comparison of the Isobaric Reagents’ Impact on Peptide Identification

We evaluated the peptide identification performance of DeAla in comparison to a well-established and cost-effective isobaric labeling reagent, DiLeu, by labeling tryptic peptides from bovine serum albumin (BSA). Both DeAla and DiLeu achieved high labeling efficiencies of approximately 99%. We then assessed their performance in terms of peptide fragmentation behavior and identification. Peptide fragmentation performance was evaluated using the XCorr value, which measures the degree of matching between fragment ions from experimental data and the theoretical fragment ions of candidate peptides from the database (Figure S4).^43^ The distribution of peptide XCorr values for tryptic peptides from BSA, labeled with either DeAla or DiLeu, and analyzed on the Exploris 480 mass spectrometer, revealed distinct patterns. DeAla-labeled peptides tend to have higher XCorr values, peaking at 2.5–3.0, compared to 1.5–2.0 for DiLeu-labeled peptides. For example, the representative spectra of the tryptic peptide LKHLVDEPQNLIK labeled with DeAla (Figure S5A) or DiLeu (Figure S5B) were collected under the optimized HCD conditions that yielded the highest numbers of peptide identifications for each tag. The spectra demonstrated higher fragmentation intensity and a greater total number of product ions with DeAla labeling. Table S1 provides a comparative list of shared peptide sequences labeled by both reagents, along with total product ions (b/y ions) and XCorr scores. Notably, 60.9% of DeAla-labeled peptides achieved higher XCorr values than their DiLeu-labeled counterparts, while 24.3% showed similar XCorr values. Additionally, higher reporter intensities in MS2 spectra can suppress peptide backbone fragmentation signals, reducing peptide and protein identifications.^33^ The reporter intensities of DiLeu labeling were approximately 60% higher than those of DeAla labeling. The superior fragmentation performance of DeAla results in an improved balance between reporter ion and peptide fragment ion abundances.

In addition to labeling BSA peptides, we extended the comparison to proteomics experiments in two cancer cell lines: MDA-MB-231 (breast cancer) and PANC-1 (pancreatic cancer). The labeling performance of DeAla was evaluated alongside DiLeu, TMT, and label-free approaches (Figure S6). In MDA-MB-231 cells, DeAla-labeled samples demonstrated comparable peptide and protein identification numbers to label-free samples and showed a 9% increase in peptide identifications compared with TMT-labeled samples. Moreover, in both cancer cell lines, DeAla labeling outperformed DiLeu labeling in terms of protein and peptide identification. Table S2 provides a list of shared tryptic peptides from MDA-MB-231 cells labeled with DeAla or DiLeu, consistent with the BSA peptide data, showing that 63.3% of DeAla-labeled peptides achieved higher XCorr values than their DiLeu-labeled counterparts. These findings suggest that DeAla offers a competitive advantage in both protein and peptide identification, resulting in better fragmentation signal quality and enabling more comprehensive protein coverage in complex biological samples such as cancer cell lines.

### Development and Optimization of a Set of 13-Plex DeAla Isobaric Tags

We expanded the multiplexing capacity of our newly designed DeAla isobaric reagents, enabling quantitative analysis with a 13-plex set (Figure 1B). This was achieved without increasing structural complexity by exploiting mass defects generated through the selective incorporation of ^13^C, ^15^N, and ^2^H stable isotopes in the reporter group.^42^ The strategic placement of deuterium atoms adjacent to nitrogen atoms was essential for minimizing chromatographic shifts between different isotopologues, ensuring consistent retention times.^31,44^ The resulting reporter isotopologues differed by mass intervals of 5.84 mDa or 6.32 mDa (Figure S7). The 13-plex DeAla reagents consisted of one 100 variant, two 101 variants, three 102 variants, three 103 variants, two 104 variants, and two 105 variants. No additional synthetic steps were required to design the 13-plex DeAla reagents, and the detailed synthetic routes for each variant are shown in Figure S8. Achieving baseline resolution of the 13 reporter ions required high resolving power in Orbitrap MS/MS acquisition. However, increasing signal acquisition time to attain higher resolving power led to fewer peptide identifications due to longer scan times. To determine the optimal resolving power for baseline resolution of the 13 reporter ions, we combined equal concentrations of each of the 13-plex DeAla reagents and infused the mixture into an Exploris 480 mass spectrometer. Using HCD-MS/MS acquisition, we evaluated resolving powers ranging from 15k to 240k (at *m*/*z* = 200), as shown in Figure 2. At a resolving power of 60k, the neighboring reporter ion peaks were distinguishable and achieved baseline separation. Therefore, an MS/MS acquisition resolution of 60k was selected as optimal for quantitative proteomics.

**Figure 1:**
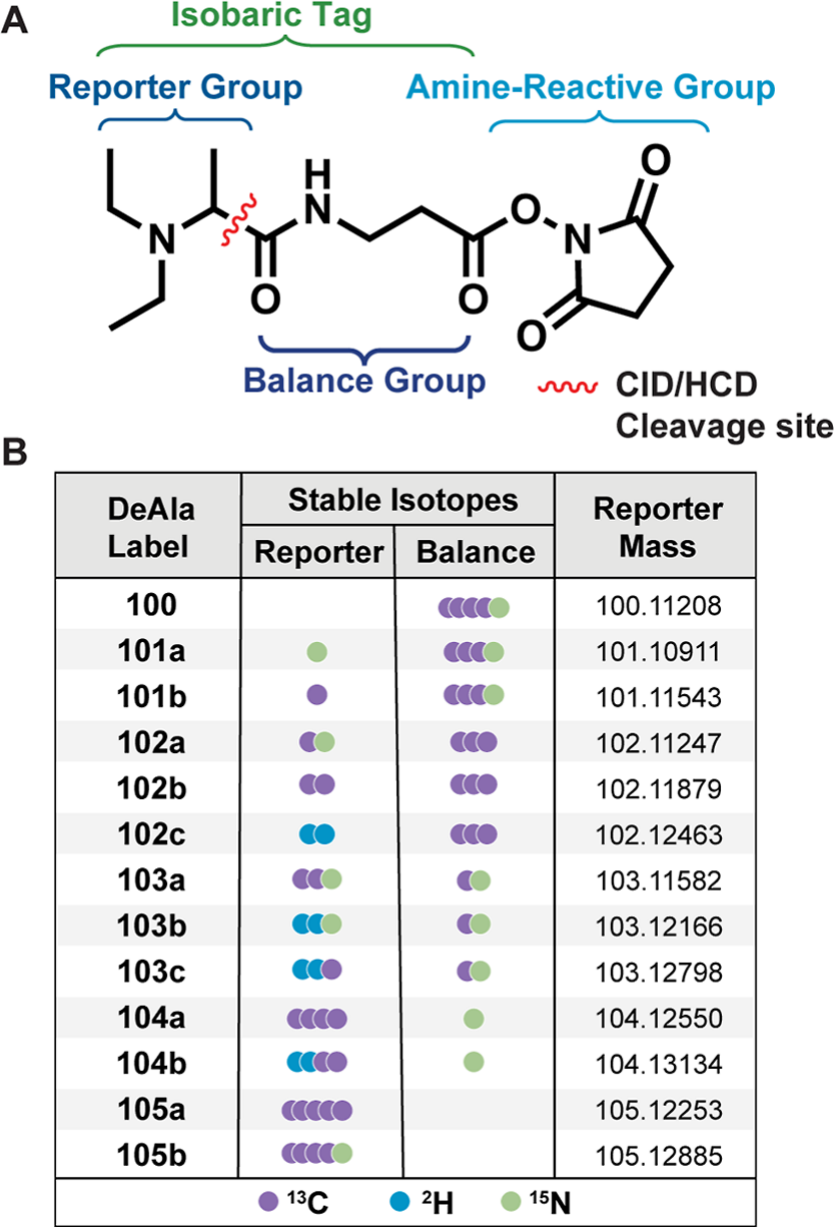
Structure and isotopic composition of the 13-plex DeAla reagents. (A) Chemical structure of the isobaric DeAla tag. The tag is designed with a CID/HCD cleavage site (highlighted in red) to enable reporter ion release during tandem mass spectrometry. (B) Table showing the isotopic composition and reporter masses for the 13-plex DeAla reagents. Each label incorporates stable isotopes (^13^C, ^2^H, ^15^N) in the reporter and balance groups.

**Figure 2:**
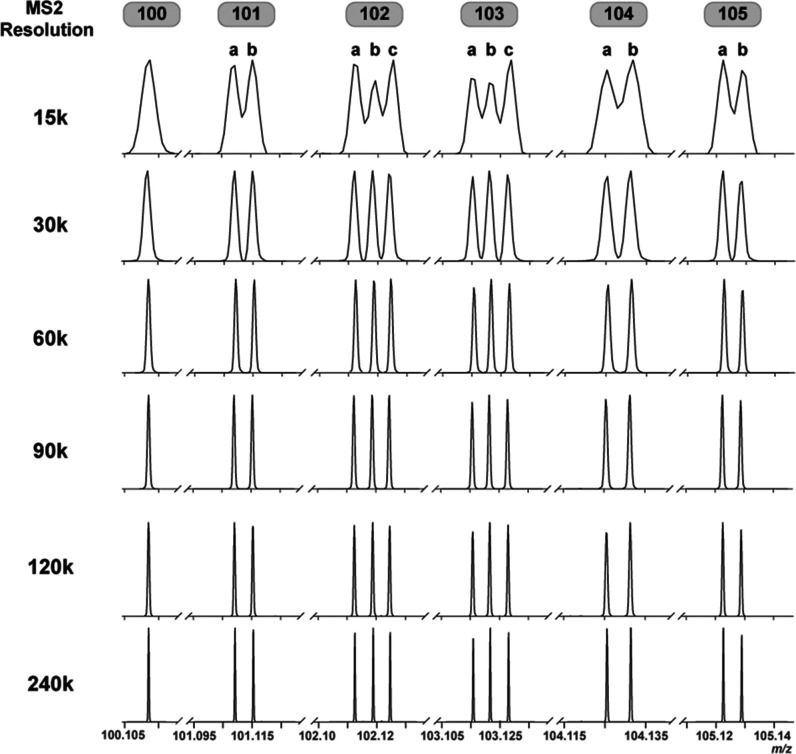
Resolution of 13-plex DeAla reporter ions. Equal concentrations of the 13-plex DeAla reagents were infused into an Orbitrap mass spectrometer. MS2 spectra of the reporter ions at resolving powers ranging from 15k to 240k are shown, with channels labeled 100 to 105.

To ensure accurate quantification, the reporter ion intensities of DeAla reagents were corrected for isotopic impurities of the individual DeAla tags during data processing. Minor isotopic impurities arose from the 98–99% purities of the isotopic starting materials used in synthesis. Each primary DeAla reporter ion peak included low-intensity impurity peaks offset by one neutron in mass. As shown in Figure S9, these impurities contributed up to 10% of the total relative isotopic abundance, while the primary ions accounted for 90–99%. The isotopic correction factors, detailed in Figure S10, adjusted for signal overlap by subtracting the estimated impurity contributions from neighboring channels. This correction was critical for accurate quantification, particularly in high-plex quantitative experiments, to minimize isotopic interference and ensure reliable quantification performance.

### Quantitative Accuracy, Replicate Variance, and Reproducibility

We assessed the quantitative accuracy and dynamic range of the 13-plex DeAla reagents by labeling MDA-MB-231 tryptic peptides and combining them in specific ratios: 1:1:1:1:1:1:1:1:1:1:1:1:1 and 1:2:4:8:12:16:8:16:12:8:4:2:1. Figure 3 illustrates the quantitative accuracy of the 13-plex DeAla reagents, where the abundance of reporter ions closely matched the expected ratios, demonstrating high accuracy in complex quantitative proteomics. The reporter ion abundances reflected the expected ratios, with average coefficients of variation (CVs) of 5.3% for the 1:1 ratio sample and 14.9% for the 16:1 ratio sample, indicating strong quantification performance. The variance and reproducibility of the 13-plex DeAla labeling approach were evaluated by comparing the quantitative ratios of identified proteins across three technical replicates of a 1:2:4:8:12:16 mixed sample. Figure 4 shows a high degree of correlation between replicates (Pearson *r* > 0.98). The log_2_ ratios between replicates aligned closely with the expected values across different runs, demonstrating excellent reproducibility over the 16-fold dynamic range. This strong correlation indicates minimal technical variability, highlighting the robustness of the 13-plex DeAla reagents in delivering accurate and reproducible quantitative results in proteomic analyses.

**Figure 3:**
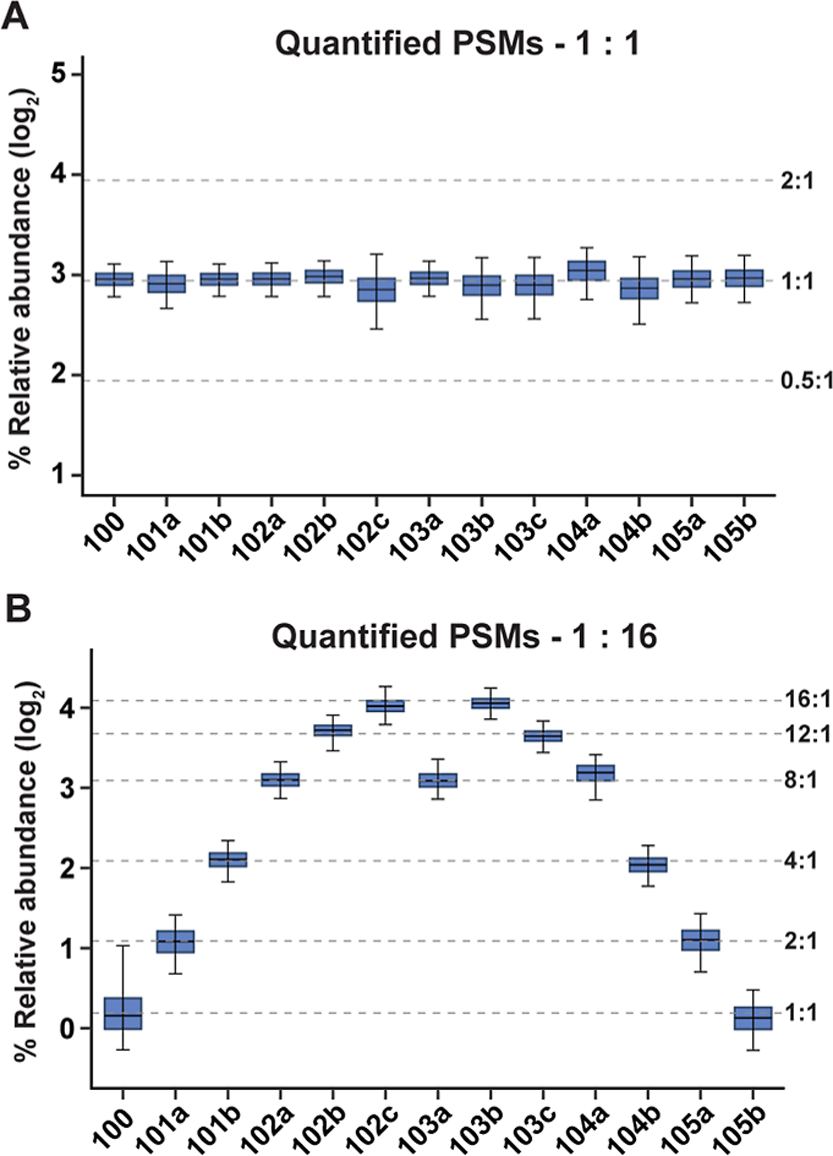
Quantitative accuracy of 13-plex DeAla labeled samples. Tryptic peptides from MDA-MB-231 cells were labeled with 13-plex DeAla reagents. The samples were combined in either 1:1:1:1 ratios or 1:2:4:8:12:16 ratios and analyzed by LC–MS/MS at a resolving power of 60k. Quantitative ratios were determined from PSMs for (A) the 1:1:1:1 ratio sample and (B) the 1:2:4:8:12:16 ratio sample. Box plots represent the median (line), the 25th and 75th percentiles (box), and the fifth and 95th percentiles (whiskers).

**Figure 4:**
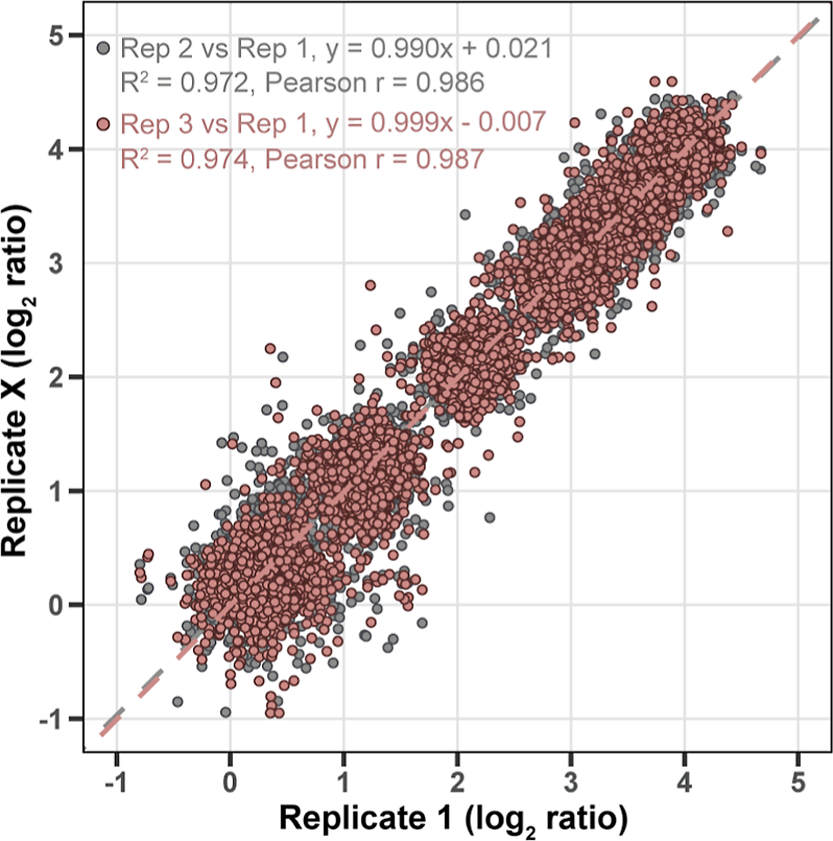
Replicate variance and reproducibility of 13-plex DeAla labeled samples. Scatter plots display the log_2_ ratios from replicate experiments. Quantitative ratios were determined from proteins identified across three technical replicates of the 1:2:4:8:12:16 ratio sample. Data points represent comparisons between replicates, with linear regression lines indicating correlation.

### Performance Comparison between 12-Plex DiLeu and 13-Plex DeAla

The newly developed 13-plex DeAla reagents were evaluated against the established 12-plex DiLeu reagents to assess their performance in quantitative proteomics. Comparisons were conducted under identical experimental conditions using tryptic peptides from MDA-MB-231 cells. As shown in Figure 5A, tryptic peptides from MDA-MB-231 cell lysates were labeled with either 13-plex DeAla or 12-plex DiLeu reagents, pooled, cleaned up by SCX, fractionated into four fractions using high-pH reversed-phase (HpH) fractionation, and analyzed via LC–MS/MS. The results indicated that DeAla labeling yielded 6,462 protein identifications and 41,512 peptide identifications, which were notably higher than those obtained with DiLeu labeling (Figure 5B). As illustrated in Figure 5C,D, the 13-plex DeAla sample identified an additional 2,021 unique proteins and 22,000 unique peptides. The overlapping identifications between the two methods included 4,441 shared proteins and 19,512 shared peptides, with 95% of proteins and 77% of peptides identified in the DiLeu sample also detected in the DeAla sample. This improvement in identification numbers suggests that the 13-plex DeAla reagents provide enhanced proteome depth in complex samples.

**Figure 5:**
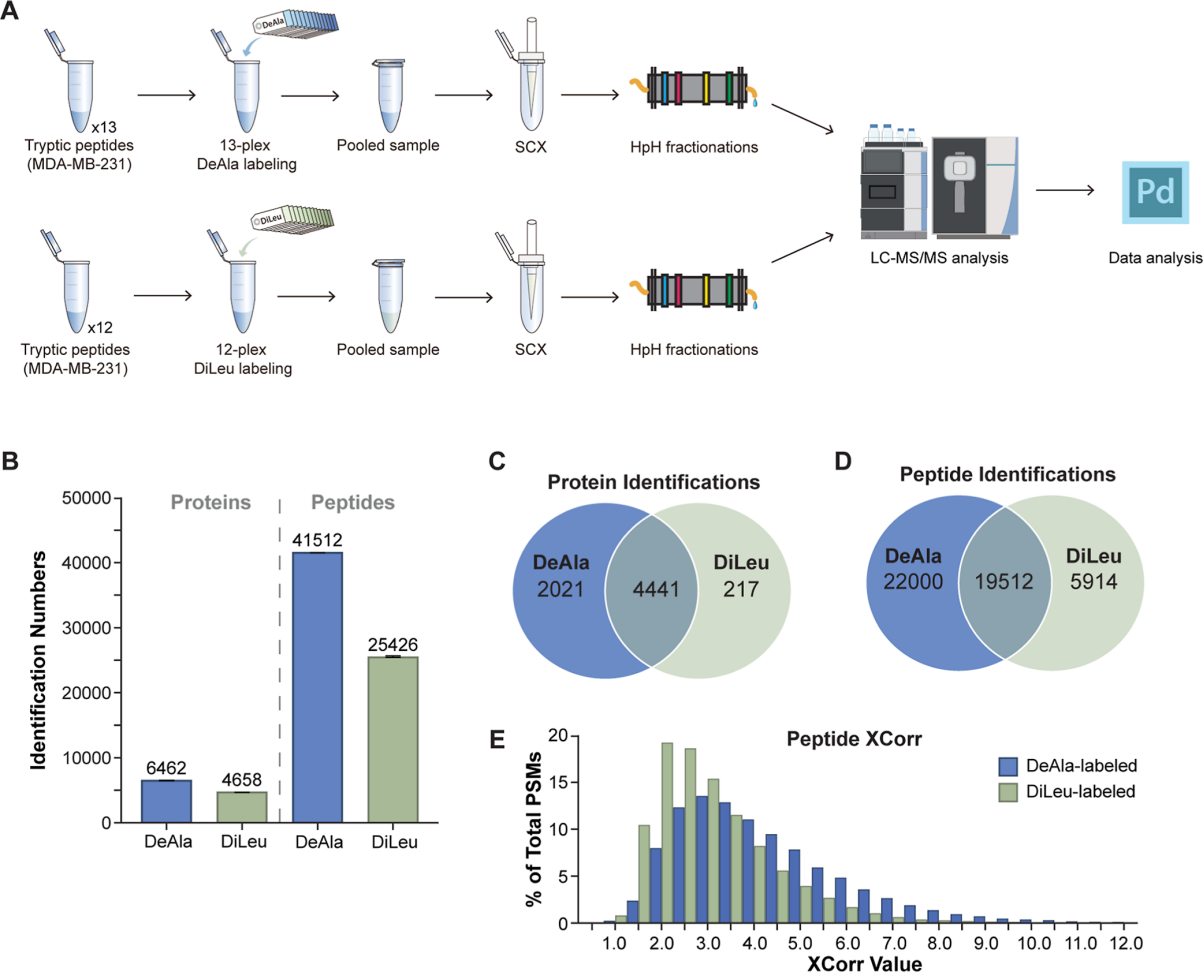
Labeling performance comparison between 13-plex DeAla and 12-plex DiLeu methods. (A) Workflow schematic for tryptic peptide labeling using the 12-plex DiLeu and 13-plex DeAla methods. (B) Bar graph showing the number of protein and peptide identifications for the DeAla and DiLeu labeling methods. (C) Venn diagram of protein identifications. (D) Venn diagram of peptide identifications. (E) XCorr distribution of tryptic peptides labeled with DeAla and DiLeu.

Beyond identification numbers, the 13-plex DeAla reagents also demonstrated superior fragmentation quality, as evidenced by the distribution of XCorr values in Figure 5E. DeAla-labeled peptides generally exhibited higher XCorr values, peaking at 2.5–3.0, in contrast to the 1.5–2.0 peak observed with DiLeu-labeled peptides. This shift toward higher XCorr values indicates increased confidence in peptide identification with DeAla reagents, likely due to their optimized ionization and fragmentation characteristics. Consequently, the improved fragmentation quality provided by DeAla is particularly advantageous for high-confidence peptide identification, which is essential in complex proteomic analyses. Taken together, these findings suggest that 13-plex DeAla reagents offer not only more comprehensive protein and peptide identification but also enhanced reliability in peptide fragmentation. Thus, DeAla reagents enable precise, high-resolution quantitation with minimal compromise in identification numbers, positioning them as a highly effective alternative to existing isobaric tagging methods.

## Conclusion

In this study, we introduced and evaluated a novel, cost-effective, alanine-based isobaric labeling reagent named DeAla. We optimized labeling efficiency and collision energy parameters, demonstrating that complete labeling is achieved with a DeAla tag-to-peptide ratio above 10:1. Our findings revealed that DeAla-labeled peptides produce more backbone fragmentation ions and higher XCorr values compared to those labeled with DiLeu, enhancing peptide identification in proteomic analyses. By incorporating stable isotopes, we expanded the multiplexing capacity of DeAla reagents to 13-plex without increasing structural complexity. The resulting reporter isotopologues, differing by mass intervals of approximately 6 mDa, allowed for baseline resolution in Orbitrap MS/MS acquisition at a resolving power of 60k. Quantitative analyses demonstrated that DeAla provides accurate and reproducible quantification across a dynamic range with minimal technical variability. Comparative proteomic analyses of MDA-MB-231 cells showed that DeAla labeling outperformed DiLeu in both protein and peptide identification numbers. The 13-plex DeAla reagents also demonstrated superior fragmentation quality, evidenced by higher XCorr values and increased confidence in peptide identification.

DeAla reagents are synthesized through a simple, high-yield, three-step process. For labeling 100 μg of protein digest per reagent in isobaric quantitative experiments, 1 mg of 13-plex DeAla reagents costs less than $25 (USD). In contrast, a 16-plex TMTpro kit containing one 5 mg vial of each reagent costs $8200 (USD). Thus, DeAla reagents are a potential multiplexing solution and an economical alternative to commercial isobaric tags, offering an accessible option for large-scale proteomic studies. For instance, 13-plex reagents enable the analysis of 12 experimental samples across 4 conditions, each with 3 biological replicates, with the 13th channel designated as a bridge channel for normalization across multiple batches.

In conclusion, DeAla reagents are cost-effective, high-performance isobaric tagging tools that enhance peptide fragmentation and protein identification. They facilitate more comprehensive proteome coverage and reliable quantification in large-scale proteomics studies while maintaining high quantification accuracy and reproducibility. DeAla reagents show great potential as versatile isobaric tags for various proteomics applications. Future developments are expected to further expand their utility in proteomics research, including disease biomarker discovery, studies of cellular responses, and protein interaction networks.

## Supplementary Material







## Notes

Detailed information on synthetic procedures, tag ratio optimization, NCE optimization, XCorr distributions, MS/MS spectra of DeAla and DiLeu, labeling performance comparison, structure of 13-plex reporter ions, isotopic purity, peak interference, and NMR spectra (PDF)
Peptide identification lists from BSA and MDA-MB-231 labeled with DeAla or DiLeu (Table S1) (XLSX)
Table S2 (XLSX)
