# Supplementary material for: 13-Plex DeAla Isobaric Reagents for High-Throughput Proteome Quantification
Source: Anal Chem. 2025 Oct 6;97(41):22643–50. doi: 10.1021/acs.analchem.5c03910 (PMC12547851; doi:10.1021/acs.analchem.5c03910)
Supplement: Supplementary file 1 [file ac5c03910_si_001.pdf]

Supporting Information

13-plex DeAla Isobaric Reagents for High-Throughput Proteome Quantification

Peng-Kai Liu[1], Ting-Jia Gu[2], Shuling Xu[2], Alexander Nassar[3], Zicong Wang[2], Hung-Yu Chiang[1], Danqing Wang[3], Lingjun Li[1,2,3,4,5*]

[1] Biophysics Graduate program, University of Wisconsin-Madison, Madison, WI, 53705, USA

[2] School of Pharmacy, University of Wisconsin-Madison, Madison, WI, 53705, USA

[3] Department of Chemistry, University of Wisconsin-Madison, Madison, WI, 53706, USA

[4] Lachman Institute for Pharmaceutical Development, School of Pharmacy, University of Wisconsin-Madison, Madison, WI, 53705, USA

[5] Wisconsin Center for NanoBioSystems, School of Pharmacy, University of Wisconsin-Madison, Madison, WI 53705, USA.

***Corresponding author:** Lingjun Li

Post address: 777 Highland Ave, Madison, Wisconsin, United States

ZIP Code: 53705

Email: lingjun.li@wisc.edu

Tel: +1 608-265-8491

Fax: +1 608-262-5345

Table of Contents

Supplemental Methods

Synthesis of DeAla reagents

Supplemental Figures

Figure S1. Synthesis scheme of DeAla reagents.

Figure S2. Optimization of DeAla-to-peptide ratios for labeling efficiency.

Figure S3. Optimization of normalized collision energy (NCE) for DeAla-labeled peptides.

Figure S4. XCorr distribution from labeled BSA tryptic peptides.

Figure S5. Representative MS/MS spectra of BSA tryptic peptides labeled with (A) DeAla and (B) DiLeu tags.

Figure S6. Labeling performance comparison across different labeling strategies.

Figure S7. 13-plex DeAla reporter ion structures.

Figure S8. 13-plex DeAla reagent syntheses

Figure S9. Measured isotopic purity fractions of reporter ion signals.

Figure S10. Isotopic peak interferences with neighboring primary reporter ion signals.

Figure S11-16. ^{1}H NMR and ^{13}C NMR spectra

Supplemental Tables (separate excel spreadsheets)

Table S1. List of shared tryptic peptides from BSA labeled with DeAla or DiLeu.

Table S2. List of shared tryptic peptides from MB-231 lysate labeled with DeAla or DiLeu.

Supplemental Methods

Synthesis of DeAla Reagents

A synthesis scheme for DeAla and the isotopic reagents are shown in the Supporting Information (Figure S1 and Figure S8). L-Alanine (**1**) (100 mg, 1.12 mmol) and $NaBH_3CN$ (183 mg, 2.91 mmol) were dissolved in 7.5 mL MeOH at 0 °C. Acetaldehyde (282 µL, 5.04 mmol) was added to the cooled solution, and the resulting reaction mixture was stirred for 16 h. The reaction mixture was then concentrated *in vacuo*, and the residue was purified by silica gel column chromatography (MeOH/DCM) to yield diethylalanine (**2**) (160 mg, 98%) as a white solid. ^1H NMR (400 MHz, CD_3OD) δ 3.78 – 3.72 (q, 1H), 3.33 – 3.24. (m, 2H), 3.16 – 3.07 (m, 2H), 1.48 – 1.46 (d, 3H), 1.35 – 1.31 (t, 6H); ^{13}C NMR (100 MHz, CD_3OD) δ 173.78, 63.28, 47.33, 12.69, 10.11; HRMS-ESI: *m/z* calcd for $C_7H_{15}NO_2H$ $[M+H]^+$ 146.1176; found 146.1175.

Diethylalanine (**2**) (160 mg, 1.10 mmol) was dissolved in 5.5 mL DMF. TSTU (298.7 mg, 0.99 mmol) and NMM (242 µL, 2.21 mmol) were added to the reaction mixture, and the reaction mixture was stirred for 1 h. Subsequently, β-alanine (112.9 mg, 1.27 mmol) was added, and the resulting reaction mixture was stirred for 6 h. The reaction mixture was concentrated *in vacuo*, and the residue was purified by silica gel column chromatography (MeOH/DCM) to yield 3-(2-(diethylamino)propanamido)propanoic acid (**3**) (190 mg, 90%) as a pale yellow solid. ^1H NMR (400 MHz, CD_3OD) δ 4.02 – 3.97 (q, 1H), 3.55 – 3.51. (m, 2H), 3.34 – 3.22 (m, 2H), 3.24 – 3.15 (m, 2H), 2.60 – 2.57 (t, 2H), 1.55 – 1.54 (d, 3H), 1.35 – 1.31 (t, 6H); ^{13}C NMR (100 MHz, CD_3OD_3) δ 173.09, 168.01, 59.72, 48.00, 43.39, 34.87, 32.38, 12.59, 8.22, 6.41; HRMS-ESI: *m/z* calcd for $C_{10}H_{20}N_2O_3H$ $[M+H]^+$ 217.1547, found 217.1546.

3-(2-(diethylamino)propanamido)propanoic acid (**3**) (50 mg, 0.23 mmol) and NHS (31.9 mg, 0.28 mmol) were dissolved in 4.6 mL DCM. DIC (43.5 µL, 0.28 mmol) was added to the solution, and the resulting reaction mixture was stirred for 2 h. The reaction mixture was then concentrated *in vacuo*, and the residue was purified by silica gel column chromatography (Acetone/Acetonitrile/DCM) to yield DeAla (**4**) (57.1 mg, 79%) as a pale yellow solid. ^1H NMR (400 MHz, CD_3CN) δ 7.28 (br, 1H), 3.88 – 3.83 (q, 1H), 3.60 – 3.56, (m, 2H), 3.15 – 3.10 (m, 2H), 3.02 – 2.97 (m, 2H), 2.90 – 2.87 (t, 2H), 2.78 – 2.74 (m, 4H), 1.96 – 1.92 (m, 3H), 1.42 – 1.40 (t, 6H); ^{13}C NMR (100 MHz, CD_3CN) δ 171.40, 168.84, 61.47, 47.30, 36.36, 31.82, 26.73, 13.19, 10.70; HRMS-ESI: m/z calcd for $C_{14}H_{23}N_3O_5H$ $[M+H]^+$ 314.1711, found 314.1708.

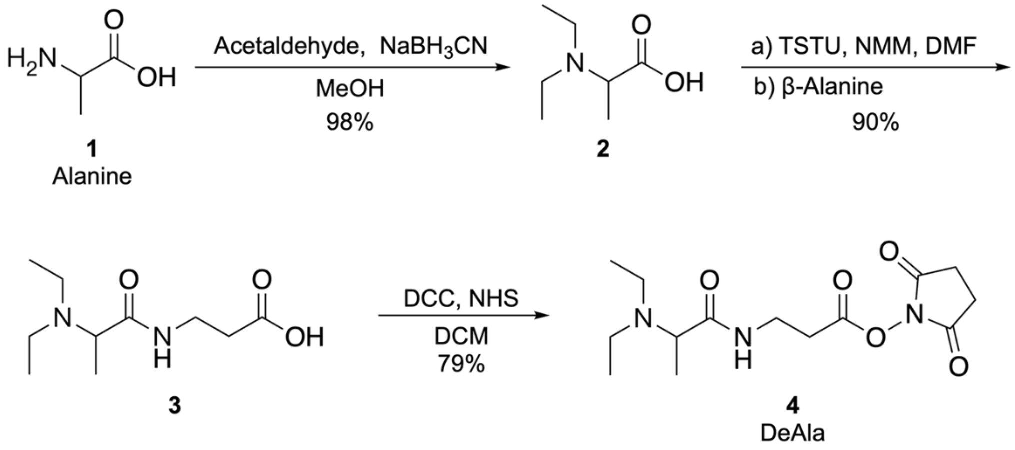

Figure S1. Synthesis scheme of the DeAla reagents. DeAla synthesis involves diethylation of alanine, β-alanine conjugation, and NHS esterification.

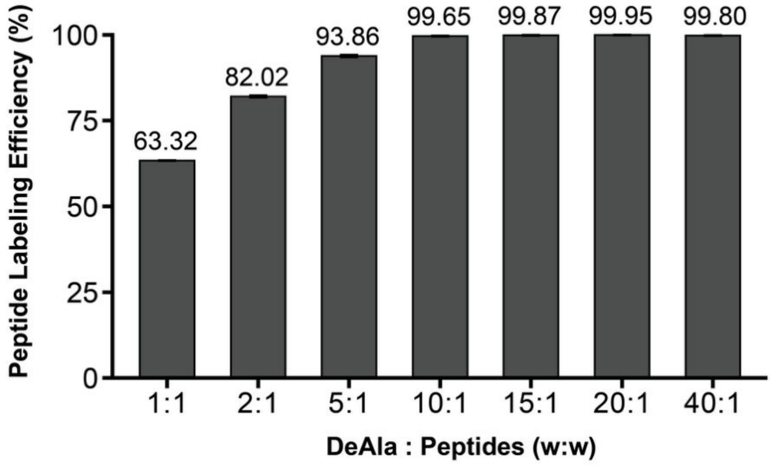

Figure S2. Optimization of DeAla-to-peptide ratios for labeling efficiency. Peptide labeling efficiency was assessed using different weight ratios (w:w) of DeAla to peptides: 1:1, 2:1, 5:1, 10:1, 15:1, 20:1, and 40:1. The MDA-MB-231 tryptic peptides were labeled with the DeAla tag. The data were acquired using a Q Exactive mass spectrometer. Error bars represent the standard deviation from duplicate experiments.

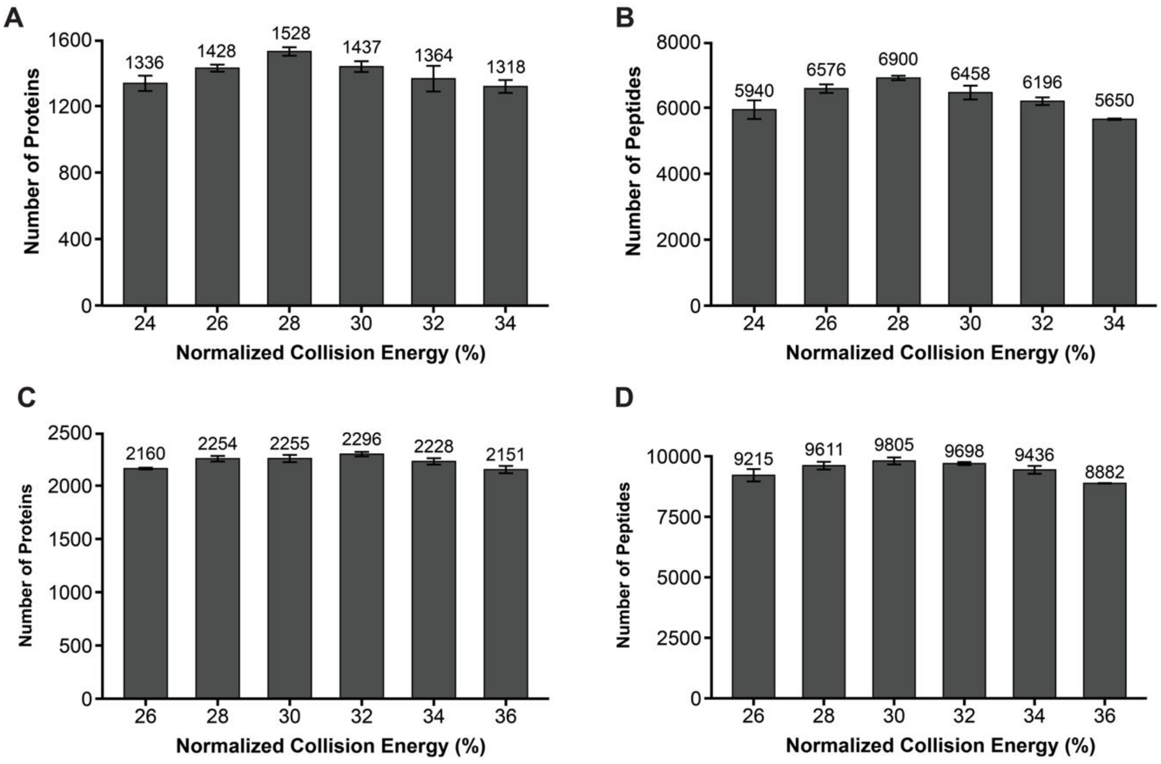

Figure S3. Optimization of normalized collision energy (NCE) for DeAla-labeled peptides. The MDA-MB-231 tryptic peptides were derivatized with the DeAla tag and analyzed under different normalized collision energy (NCE) settings. (A) The number of identified proteins and (B) the number of identified peptides were obtained using a Q Exactive mass spectrometer. (C) The number of identified proteins and (D) the number of identified peptides were obtained using an Exploris 480 orbitrap mass spectrometer. Error bars represent the standard deviation from duplicate experiments.

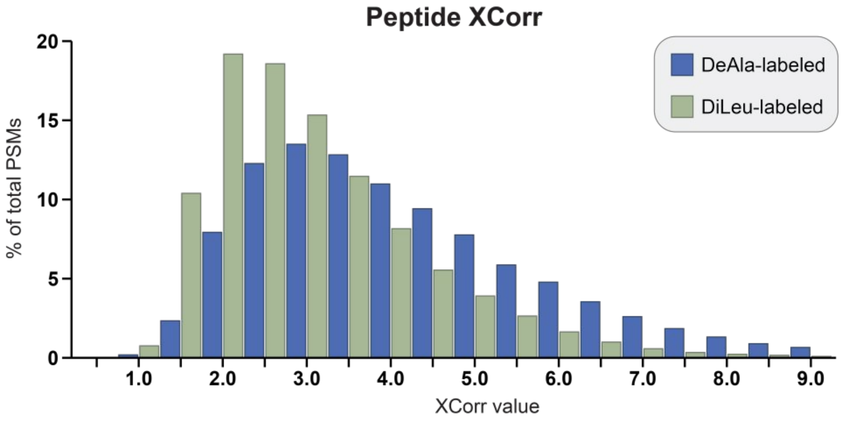

Figure S4. XCorr distribution from labeled BSA tryptic peptides. The XCorr distribution of peptide-spectrum matches (PSMs) from BSA tryptic peptides labeled with either DeAla or DiLeu tags. XCorr values were obtained from Thermo Proteome Discoverer software.

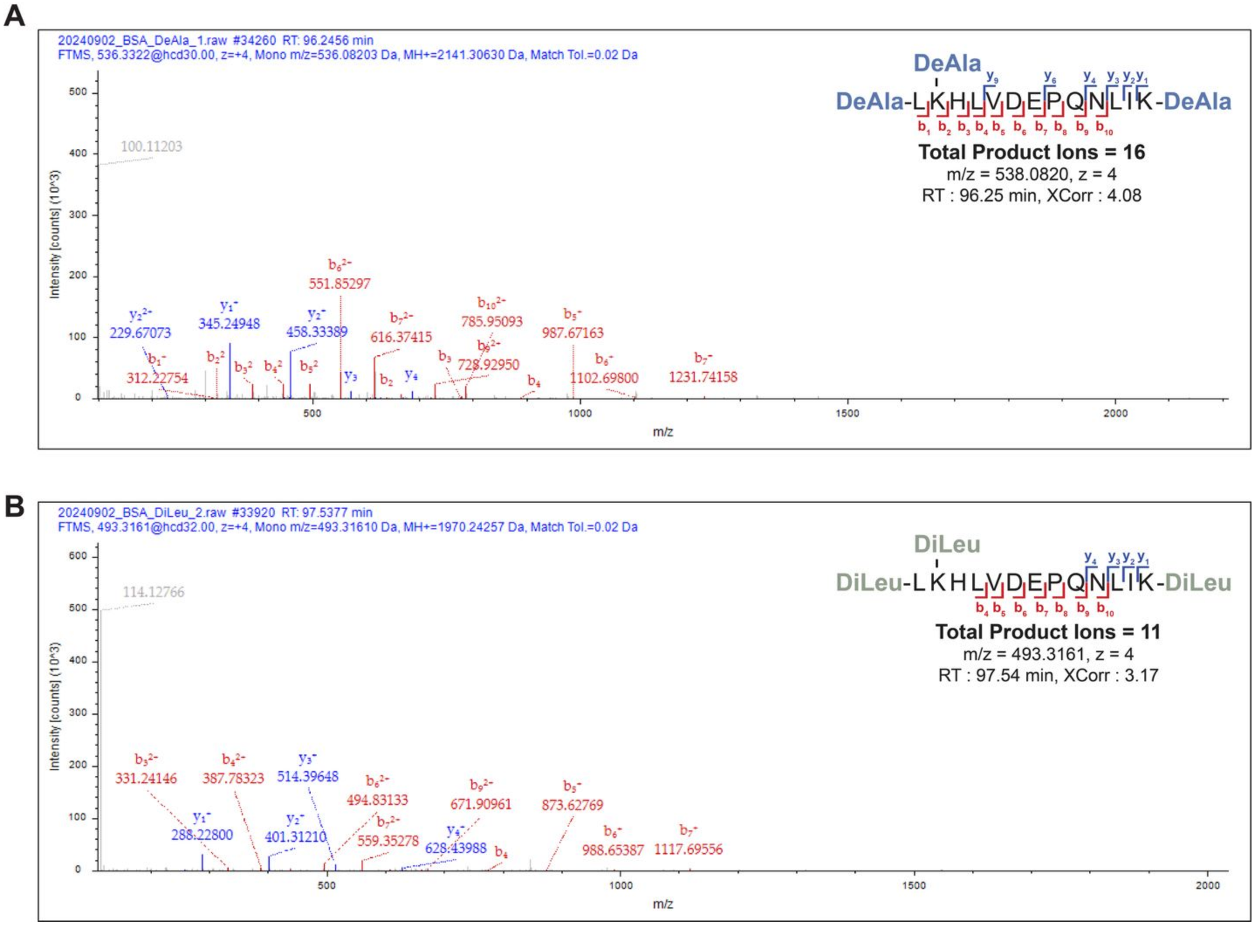

Figure S5. Representative MS/MS spectra of BSA tryptic peptides labeled with (A) DeAla and (B) DiLeu tags. Fragmentation ions include b-ions (red) and y-ions (blue), as indicated in the spectra. The total number of product ions, retention time (RT), precursor *m/z*, and XCorr score are annotated from Thermo Proteome Discoverer software.

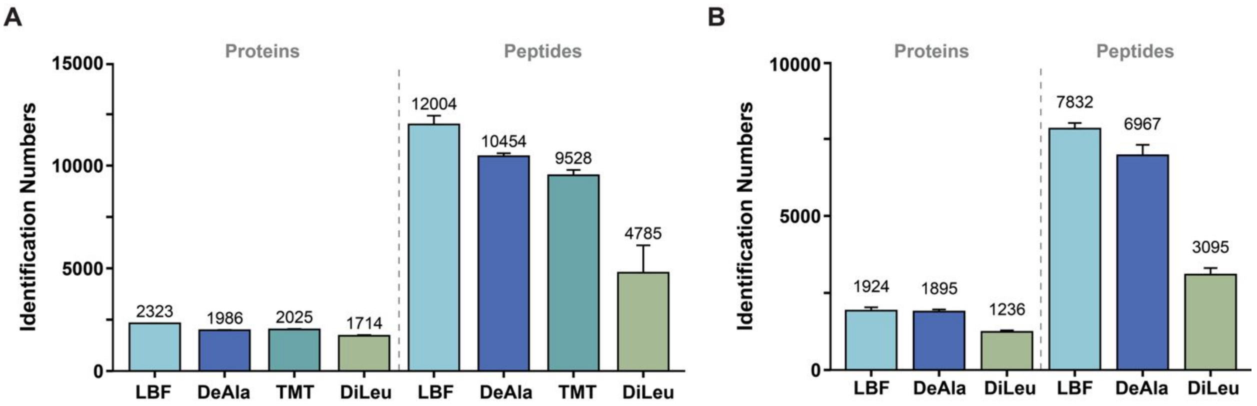

Figure S6. Labeling performance comparison across different labeling strategies. Protein and peptide identification numbers were compared using (A) label-free (LBF), DeAla, TMT and DiLeu labeling in MDA-MB-231 breast cancer cells and (B) LBF, DeAla and DiLeu labeling in PANC-1 pancreatic cancer cells. Error bars represent the standard deviation from duplicate experiments.

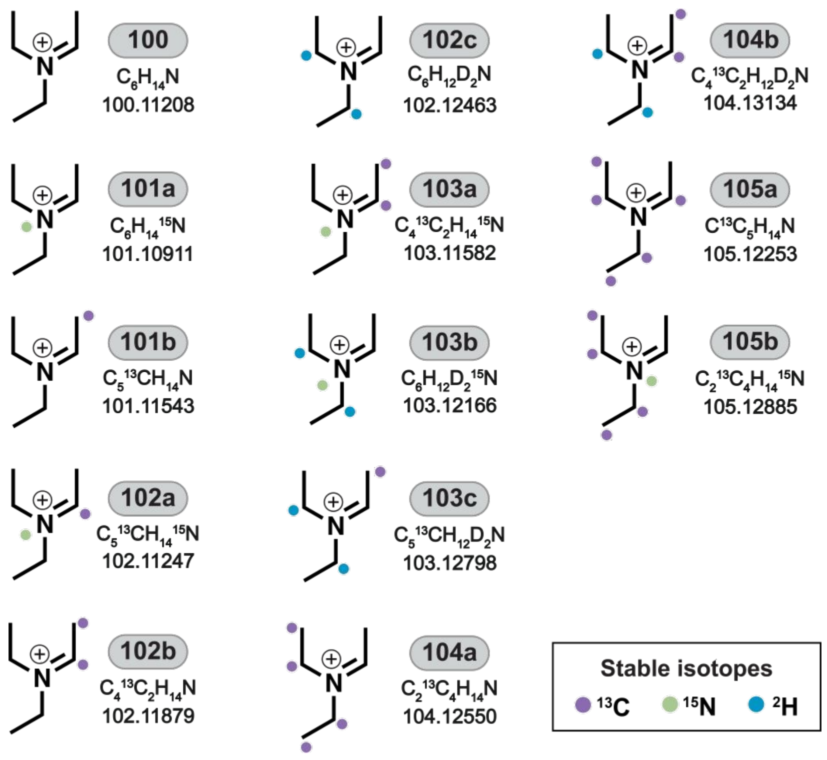

Figure S7. 13-plex DeAla reporter ion structures. The stable isotope positions of the 13 reporter ions from the DeAla reagents are shown.

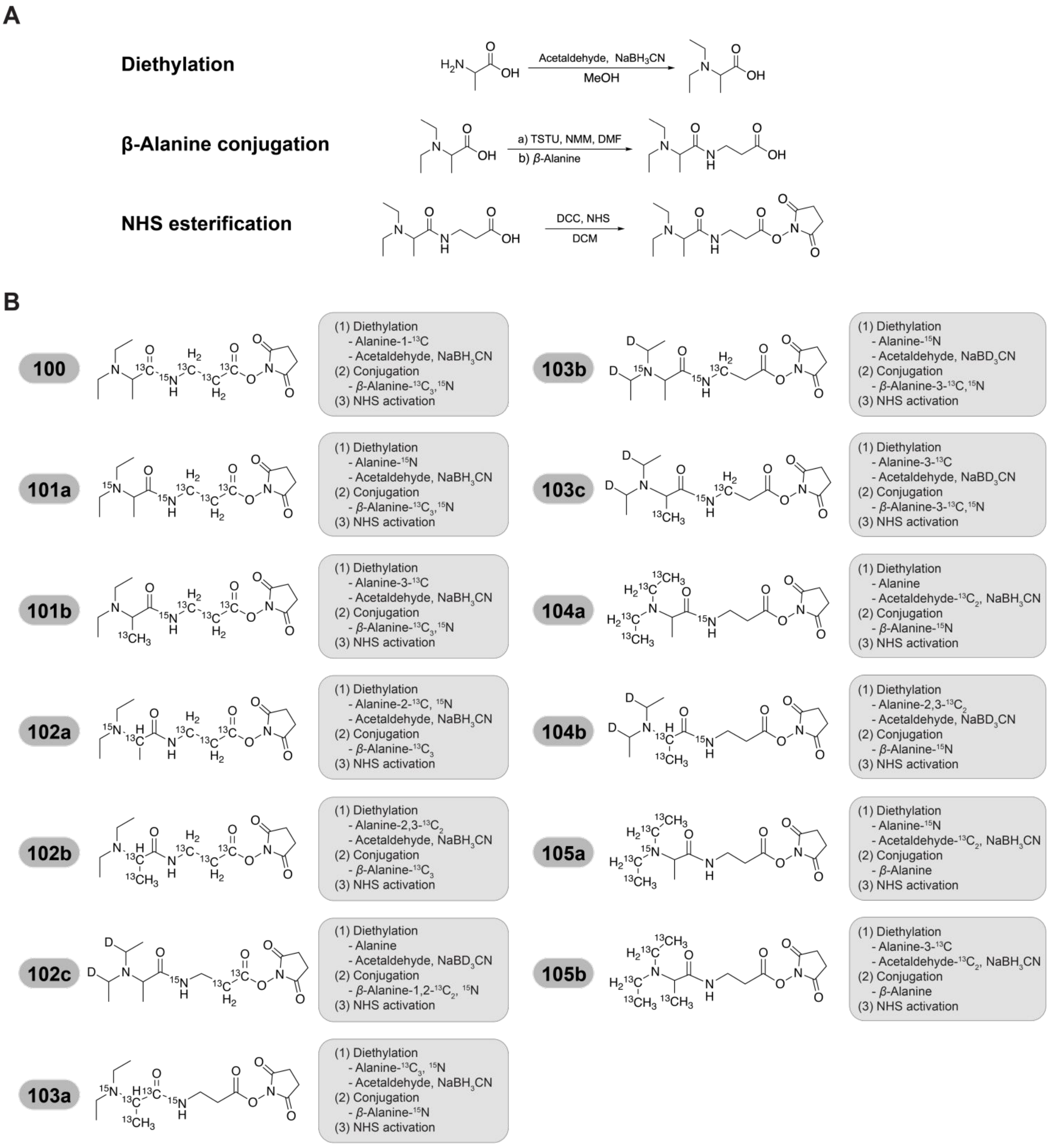

Figure S8. 13-plex DeAla reagent syntheses. (A) A schematic overview of the synthetic steps for the DeAla reagent. (B) Structures of the 13 DeAla reagents and detailed reaction steps for each reagent.

	100	101a	101b	102a	102b	102c	103a	103b	103c	104a	104b	105a	105b
-1(1H)						4.30%<br>101.11835		2.09%<br>102.11538	3.25%<br>102.1217		6.58%<br>103.12506		
-1(13C)			2.19%<br>100.11208		1.44%<br>101.11544					0.79%<br>103.12215	0.65%<br>103.12799	1.10%<br>104.11918	1.22%<br>104.1255
-1(15N)				0.55%<br>101.11544									
0	95.69%<br>100.11208	91.47%<br>101.10911	90.83%<br>101.11543	95.51%<br>102.11247	93.28%<br>102.11879	91.05%<br>102.12463	97.82%<br>103.11582	93.12%<br>103.12166	94.87%<br>103.12798	98.79%<br>104.1255	89.83%<br>104.13134	98.70%<br>105.12253	98.78%<br>105.12885
+1(13C)	3.04%<br>101.11543	8.53%<br>102.11246	6.98%<br>102.11878	3.89%<br>103.11582	5.28%<br>103.12214	4.65%<br>103.12798	2.06%<br>104.11917	4.79%<br>104.12501	1.88%<br>104.13133	0.41%<br>105.12885	2.94%<br>105.13469	0.19%<br>106.12588	
+1(15N)	1.27%<br>101.10911												

Figure S9. Measured isotopic purity fractions of reporter ion signals. The total reporter ion intensity for each channel is distributed across the primary reporter ion peak (0) and the isotopic peaks (±1) within a 20 ppm mass tolerance. Only isotopic peaks contributing more than 0.1% of the total signal are included in the data.

Primary and isotopic fractional signals

Primary signal + interferences (rows)

	100	101a	101b	102a	102b	102c	103a	103b	103c	104a	104b	105a	105b
100	95.69%		2.19%										
101a		91.47%		0.04%									
101b	3.04%		90.83%	0.55%	1.44%								
102a		8.53%		95.51%			0.12%						
102b			6.98%		93.28%								
102c						91.05%							
103a				3.89%			97.82%						
103b					5.28%			93.12%		0.79%			
103c						4.65%			94.87%		0.65%		
104a								4.79%		98.79%			1.22%
104b									1.88%		89.83%		
105a												98.70%	
105b										0.41%			98.78%

Figure S10. Isotopic peak interferences with neighboring primary reporter ion signals. The fractional intensities of the +1 and -1 isotopic peaks for each channel (columns) overlap with and contribute to the measured intensities of neighboring primary reporter ion signals (rows).

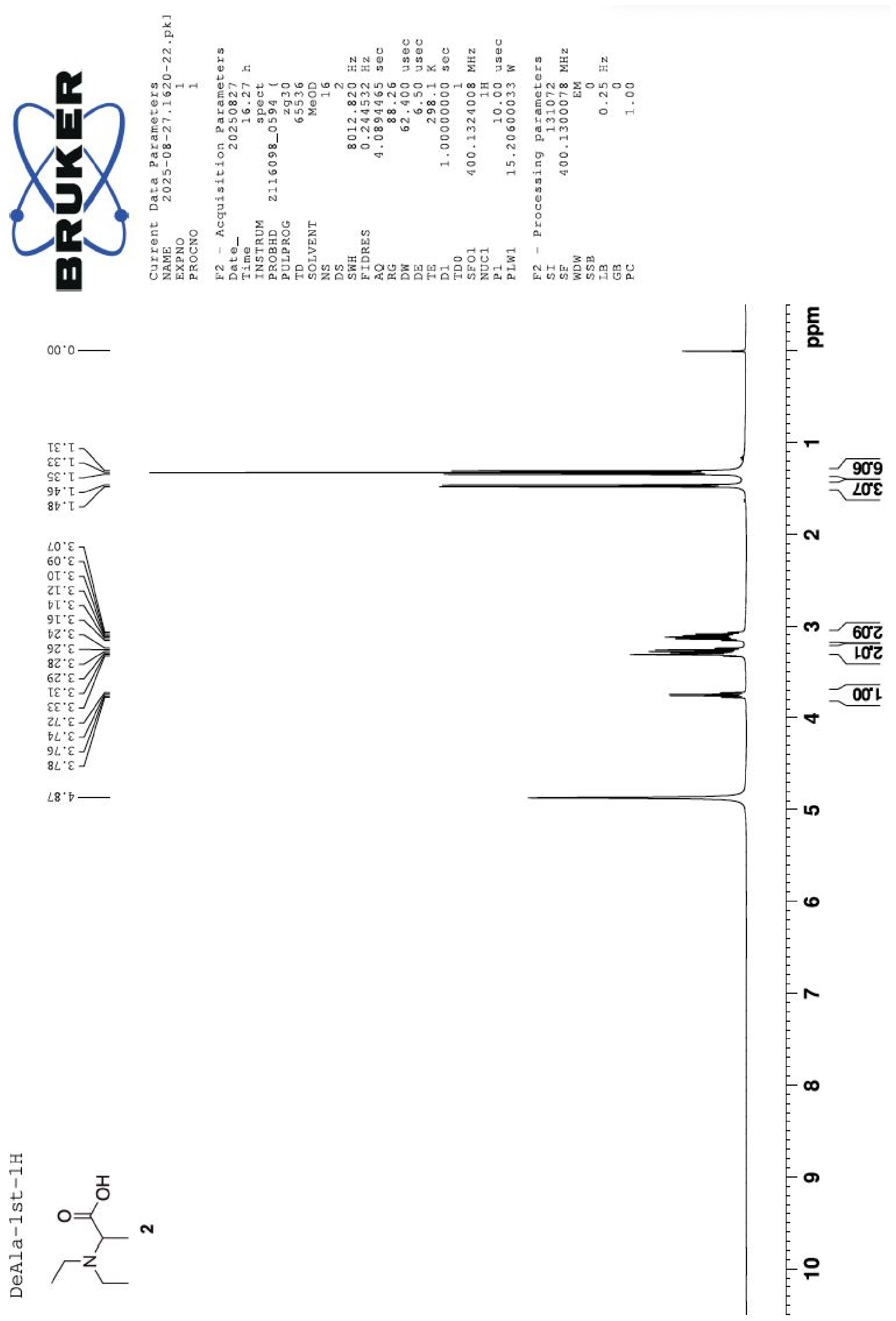

Figure S11. ^{1}H NMR spectrum of diethylalanine (2) (400 MHz, CD_3OD).

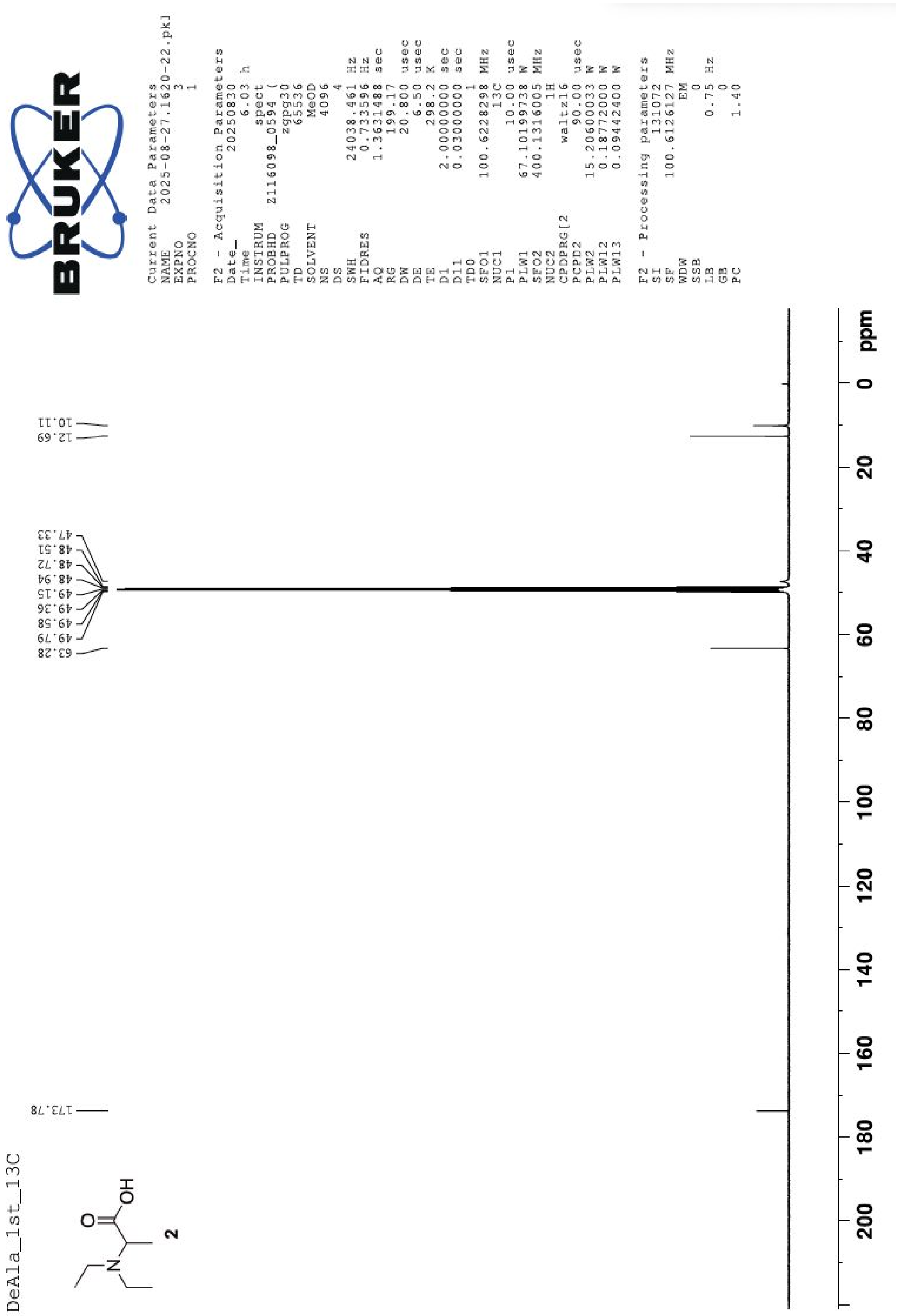

Figure S12. ^{13}C NMR spectrum of diethylalanine (2) (100 MHz, CD_3OD).

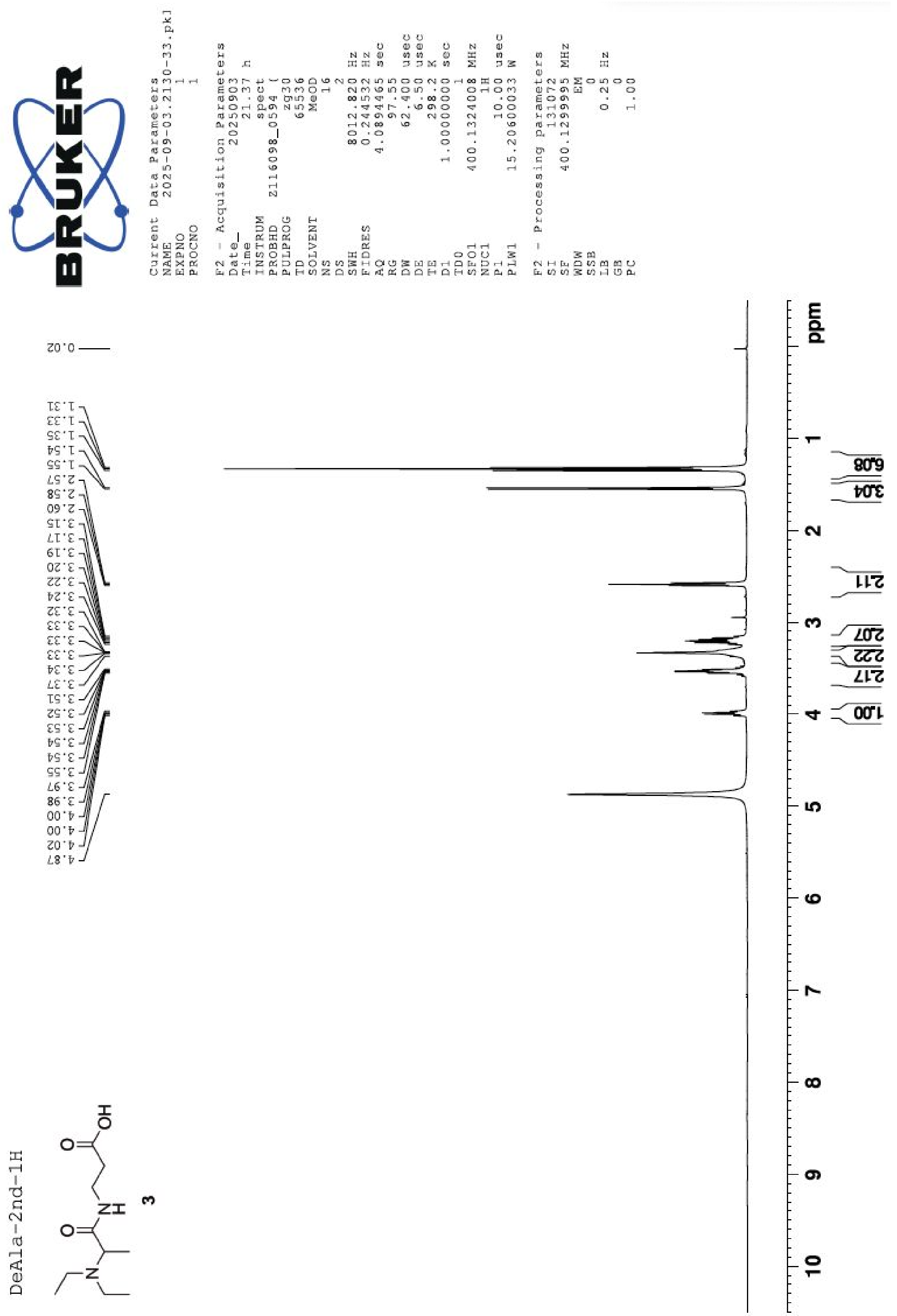

Figure S13. **^{1}H NMR spectrum of 3-(2-(diethylamino)propanamido)propanoic acid (3)** (400 MHz, CD_3OD).

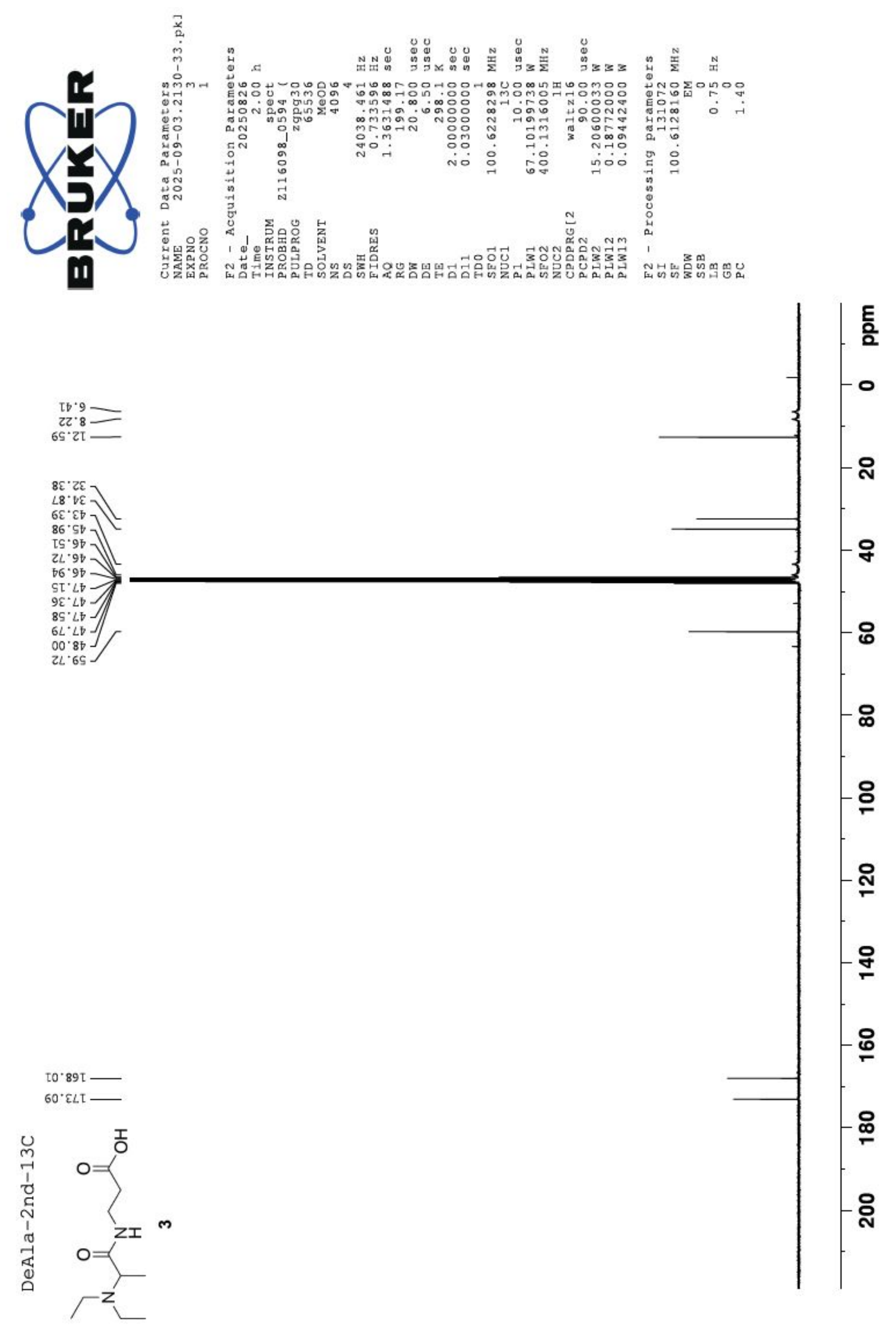

Figure S14. **^{13}C NMR spectrum of 3-(2-(diethylamino)propanamido)propanoic acid (3)** (100 MHz, CD_3OD).

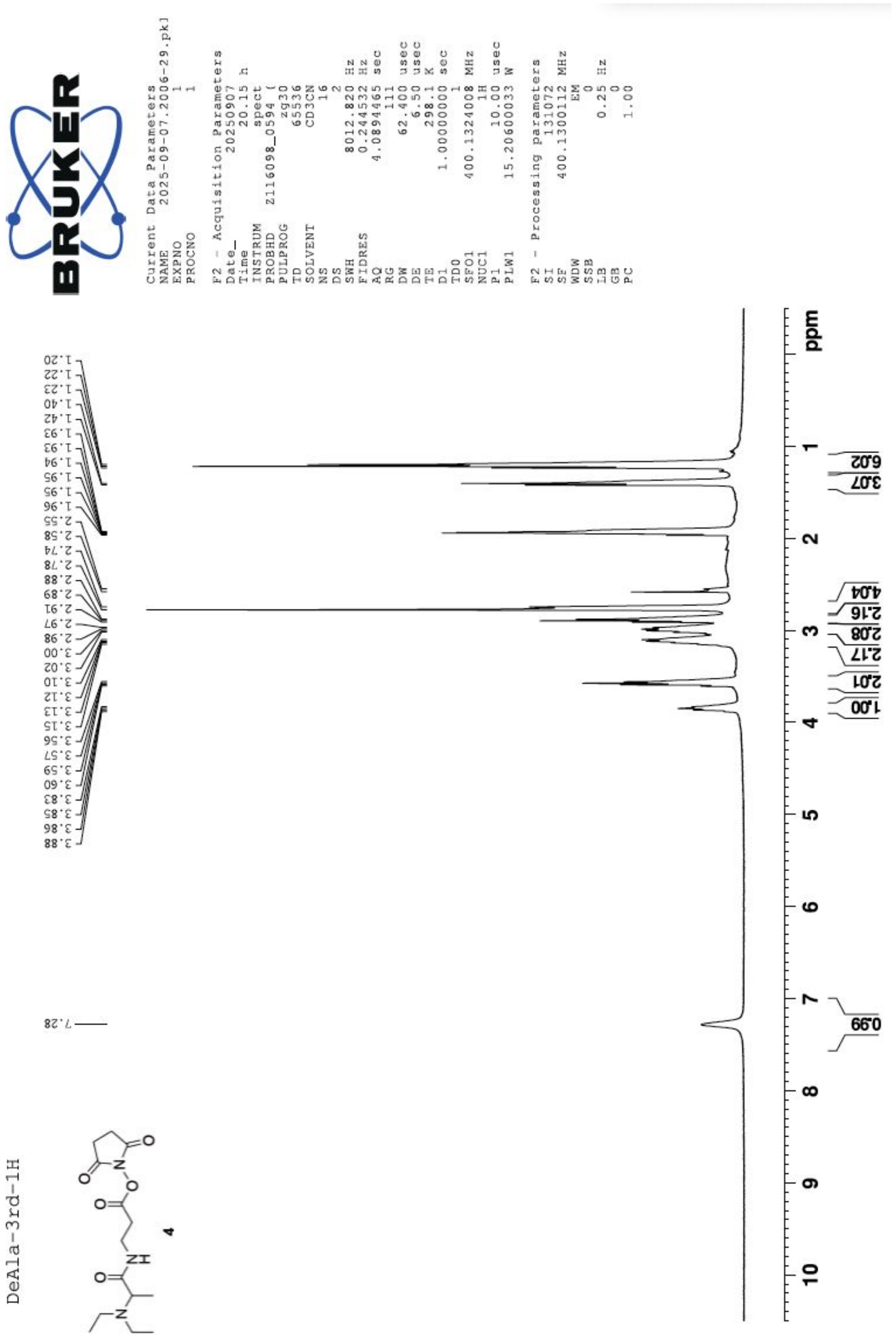

Figure S15. ^{1}H NMR spectrum of DeAla (4) (400 MHz, CD_3CN).

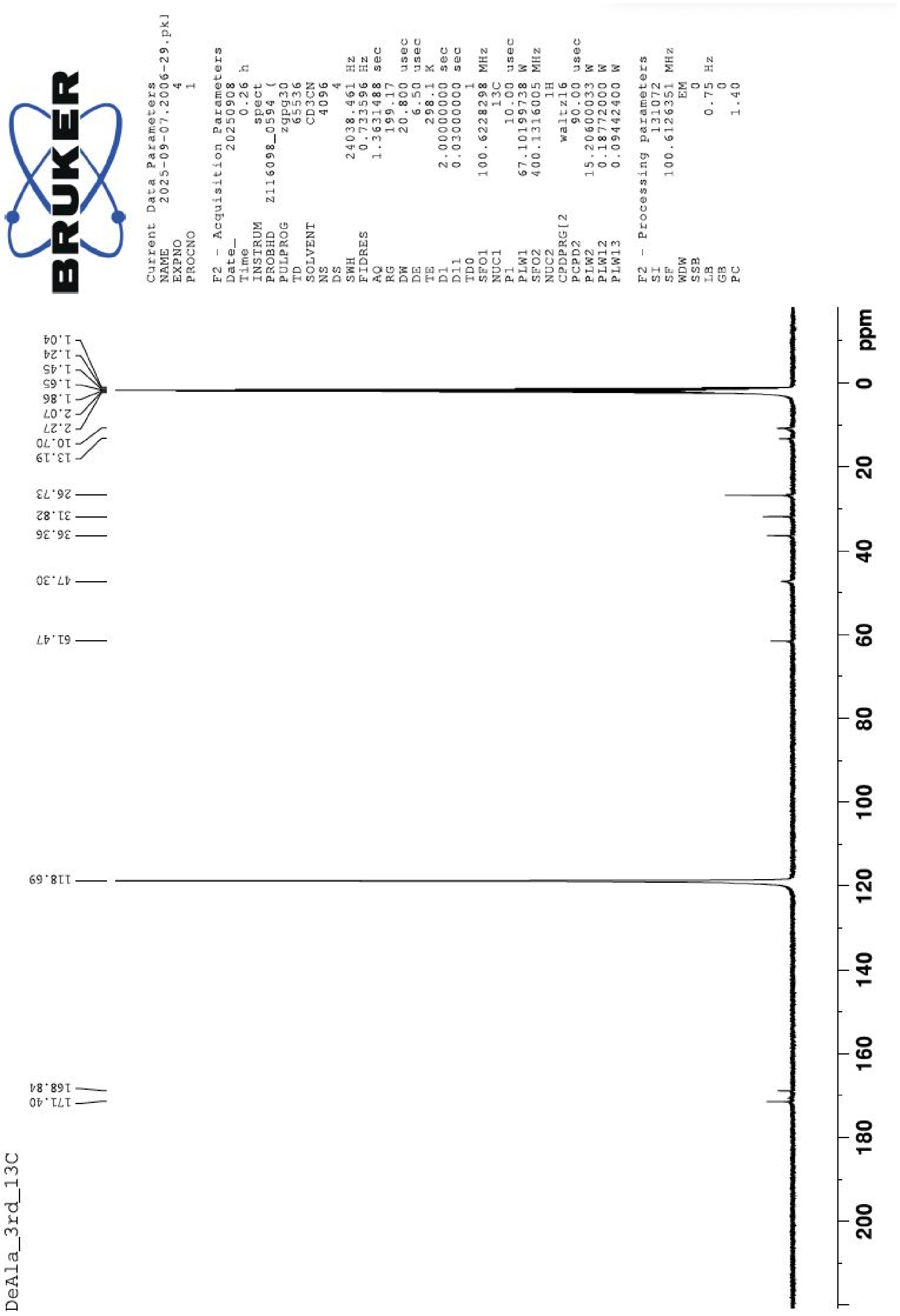

Figure S16. ^{13}C NMR spectrum of DeAla (4) (100 MHz, CD_3CN).

Table S1. List of shared tryptic peptides from BSA labeled with DeAla or DiLeu. Annotated Sequence and XCorr values were obtained from Thermo Proteome Discoverer software. Please see separate Excel spreadsheet.

Table S2. List of shared tryptic peptides from MB-231 cell lysates labeled with DeAla or DiLeu. Annotated Sequence and XCorr values were obtained from Thermo Proteome Discoverer software. Please see separate Excel spreadsheet.
